# Is mass drug administration against lymphatic filariasis required in urban settings? The experience in Kano, Nigeria

**DOI:** 10.1371/journal.pntd.0006004

**Published:** 2017-10-11

**Authors:** Dung D. Pam, Dziedzom K. de Souza, Susan D'Souza, Millicent Opoku, Safiya Sanda, Ibrahim Nazaradden, Ifeoma N. Anagbogu, Chukwu Okoronkwo, Emmanuel Davies, Elisabeth Elhassan, David H. Molyneux, Moses J. Bockarie, Benjamin G. Koudou

**Affiliations:** 1 Applied Entomology and Parasitology Unit, Department of Zoology, University of Jos, Jos, Nigeria; 2 Parasitology Department, Noguchi Memorial Institute for Medical Research, University of Ghana, Legon, Ghana; 3 Sightsavers International, UK Office, London, United Kingdom; 4 Sightsavers International, Nigeria Office, Kaduna, Nigeria; 5 NTD Division, Federal Ministry of Health, Abuja, Nigeria; 6 Centre for Neglected Tropical Diseases and Department of Parasitology, Liverpool School of Tropical Medicine, Liverpool, United Kingdom; 7 European and Developing Countries Clinical Trials Partnership, Africa Office, Cape Town, South Africa; 8 UFR Science de la Nature, Université Nangui Abrogoua, Abidjan, Cote d’Ivoire; Erasmus MC, NETHERLANDS

## Abstract

**Background:**

The Global Programme to Eliminate Lymphatic Filariasis (GPELF), launched in 2000, has the target of eliminating the disease as a public health problem by the year 2020. The strategy adopted is mass drug administration (MDA) to all eligible individuals in endemic communities and the implementation of measures to reduce the morbidity of those suffering from chronic disease. Success has been recorded in many rural endemic communities in which elimination efforts have centered. However, implementation has been challenging in several urban African cities. The large cities of West Africa, exemplified in Nigeria in Kano are challenging for LF elimination program because reaching 65% therapeutic coverage during MDA is difficult. There is therefore a need to define a strategy which could complement MDA. Thus, in Kano State, Nigeria, while LF MDA had reached 33 of the 44 Local Government Areas (LGAs) there remained eleven ‘urban’ LGAs which had not been covered by MDA. Given the challenges of achieving at least 65% coverage during MDA implementation over several years in order to achieve elimination, it may be challenging to eliminate LF in such settings. In order to plan the LF control activities, this study was undertaken to confirm the LF infection prevalence in the human and mosquito populations in three urban LGAs.

**Methods:**

The prevalence of circulating filarial antigen (CFA) of *Wuchereria bancrofti* was assessed by an immuno-chromatography test (ICT) in 981 people in three urban LGAs of Kano state, Nigeria. Mosquitoes were collected over a period of 4 months from May to August 2015 using exit traps, gravid traps and pyrethrum knock-down spray sheet collections (PSC) in different households. A proportion of mosquitoes were analyzed for *W*. *bancrofti*, using dissection, loop-mediated isothermal amplification (LAMP) assay and conventional polymerase chain reaction (PCR).

**Results:**

The results showed that none of the 981 subjects (constituted of <21% of children 5–10 years old) tested had detectable levels of CFA in their blood. Entomological results showed that *An*. *gambiae* s.l. had *W*. *bancrofti* DNA detectable in pools in Kano; *W*. *bancrofti* DNA was detected in between 0.96% and 6.78% and to a lesser extent in *Culex* mosquitoes where DNA was detected at rates of between 0.19% and 0.64%. DNA analysis showed that *An*. *coluzzii* constituted 9.9% of the collected mosquitoes and the remaining 90.1% of the mosquitoes were *Culex* mosquitoes.

**Conclusion:**

Despite detection of *W*. *bancrofti* DNA within mosquito specimens collected in three Kano urban LGAs, we were not able to find a subject with detectable level of CFA. Together with other evidence suggesting that LF transmission in urban areas in West Africa may not be of significant importance, the Federal Ministry of Health advised that two rounds of MDA be undertaken in the urban areas of Kano. It is recommended that the prevalence of *W*. *bancrofti* infection in the human and mosquito populations be re-assessed after a couple of years.

## Author summary

Mass drug administration (MDA) for the control of elephantiasis in the state of Kano in Nigeria, started in the year 2010. It was estimated that by 2015, the MDA programme will be extended to 11 remaining urban Local Government Areas (LGAs). However, MDA in urban areas faces specific challenges, the most prominent being the need to achieve coverage rates of 65% and above. As such MDA alone may not be sufficient to achieve the required programme impacts of reducing LF transmission to levels below which transmission cannot be sustained, and additional interventions may be required. This study set out to confirm the LF infection prevalence in the human and mosquito populations in three urban LGAs in Kano. Individuals were tested for signs of the disease, and mosquito samples were collected and also tested for the worms that cause the disease. The study revealed that of 981 people tested, none had circulating filarial antigen in the blood. However, the mosquitoes collected revealed the presence of the disease-causing worms, but the level of infection was low. The infection in the mosquitoes was also detected in two different types of mosquitoes. Based on the outcomes of this study, and evidence from other West African cities on the transmission of LF, the Federal Ministry of Health recommended that two rounds of MDA be undertaken in urban areas of Kano. A further reassessment after a couple of years is warranted.

## Introduction

Lymphatic filariasis (LF) is a disease caused by Nematode worms that live in the lymphatic vessels of humans. There are three species of filarial parasites which infect humans—*Wuchereria bancrofti*, *Brugia malayi* and *Brugia timori*. In Africa, LF is caused by *W*. *bancrofti* and is transmitted by *Anopheles* mosquitoes in rural settings and *Anopheles* and culicine mosquitoes in urban areas [1,2]. LF is a significant health problem in many developing countries with about 1.4 billion people known to live in endemic areas, with one quarter potentially infected [3]. LF is also the second leading cause of permanent disability and undermines the social and economic welfare of affected people and communities [4]. The World Health Assembly passed a resolution in 1997 to eliminate LF as a public health problem by the year 2020. The World Health Organization has since 2000 launched a Global Programme to Eliminate Lymphatic Filariasis (GPELF) [5]. Annual mass treatment with single-dose diethylcarbamazine (DEC) or ivermectin (IVM) in combination with albendazole (ALB) for 4–6 years is the principal tool of the elimination strategy. This has resulted in dramatic progress in reducing the prevalence of LF in many areas around the world through MDA [6].

The problem of urban LF remains a matter of debate, yet it is recognized as being a key challenge to the ongoing global efforts to eliminate LF as a public health problem [7]. This is, in part, due to the challenges of translating MDA procedures that were developed for rural areas to the urban environment, with its distinctive patterns of human organization and behaviour, and in part due to the proliferation of potential urban vectors in poorly planned urban settlements [2]. In West Africa where *Anopheles* mosquitoes are the main vectors of LF and *Culex* plays little, if any, role in LF transmission, the status of urban transmission remains ill- defined as studies of LF transmission in cities in Sierra Leone and Guinea found no evidence of transmission [8,9]. Given the rapid expansion of urban centres in West Africa over recent decades, driven by population increases, insecurity and rural-urban migration the policy for treatment of these populations needs to be defined based on the evidence that transmission is ongoing and is likely to be sustained.

In the State of Kano, Nigeria, LF MDA started in 2010 and as of 2014, had reached 33 of the 44 Local Government Areas (LGAs) known to be LF endemic. Under the plans for 2015 LF MDA was to be extended to the remaining 11 LGAs including, six ‘urban’ LGAs. In 2010, baseline ICT prevalence surveys conducted in these six urban LGAs revealed high ICT prevalence rates between 2 and 22%. Given such high pre-control endemicity levels added to the challenges of MDA implementation in urban settings, it may be useful to confirm the prevalence rates before embarking on MDA. The main goal of this study was to verify previous survey results carried out in Kano urban settings which suggested that LF is endemic. Entomological surveys were then conducted to determine the intensity of ongoing transmission in large cities in the light of recent findings that LF transmission is not sustainable in the capitals of Sierra Leone [8] and Guinea [9] despite the presence of antigen positives.

## Materials and methods

### Ethics statement

Ethical Clearance (MOH/OFF/797/Ti/15) was given by the Kano State Ministry of Health Ethical Committee. Additionally, all surveys were conducted in accordance with the study protocol approved by the Institutional Ethics Review Board of the Liverpool School of Tropical Medicine (1189RS). All levels of leadership in the three Local Government Areas used for this study were met and their approval of the work was received. Written informed consent was obtained from all study participants above the age of 18 years, as well as parental consent from children below the age of 18 years. The children below 18 years provided oral consent for the study.

### Study area and sites

Selection of study communities was based on a previous mapping by the Federal Ministry of Health, which indicated that urban LGAs were endemic for LF in Kano State. The mapping survey was undertaken in 2010, following WHO recommendations [10]. Communities were selected in LGAs, based on key informant identification of communities most likely to be endemic for filariasis. Following community sensitization, 50–100 consenting volunteers were invited to a designated place where they were examined for *W*. *bancrofti* antigenemia using the ICT cards. The results of the mapping survey are presented in Table 1. The current study was carried out in three of the urban LGAs included in the mapping survey (Ungogo, Fagge and Nasarawa). The LGAs and study communities were randomly selected among those having the lowest and the highest antigenemia rates recorded during the mapping survey. However, in Nasarawa LGA Goji, which was peri-urban, was replaced by Gama (an urban community). Jaba in Fagge LGA and Dankukuru in Ungogo LGA were also urban communities. In these LGAs neither LF MDA nor MDA for onchocerciasis based on ivermectin has been previously conducted. Kano State is located in Northern Nigeria, on latitude 11^0^ 30′ N—11^0^ 50′ N and longitude 8^0^ 30′ E—8^0^ 50′ E. The state is bound by four other states, all of which are endemic for LF- Katsina State to the North-west, Jigawa State to the North-east, Kaduna State to the South-west and Bauchi State on the South-eastern border. The status of Kano State as an LF endemic state has previously been established as a 1.6% prevalence was found in some villages [11].

**Table 1:** Results of 2010 mapping survey in Kano state.

IU	Locality	No. Examined	No. Positives	ICT Prevalence (%)
Ajingi	Ungu War Bai	47	7	14.9
Albasu	Faragai	50	1	2.0
Bagwai	Rimin Dako	50	5	10.0
Bebeji	Ran Tan	45	2	4.4
Bichi	YanaIami	45	1	2.2
Bunkure	Bunkure	50	1	2.0
Dala	DaIa	50	6	12.0
Dambatta	Ajumawa	47	2	4.3
Dawakin-Kudu	Kogar Kaza	50	5	10.0
Dawakin-Tofa	Sarauniya	47	3	6.4
Doguwa	Natsohuwa	49	5	10.2
Fagge	Jaba	50	2	4.0
Gabasawa	Gunduwa	46	6	13.0
Garko	Kafinchiri	50	2	4.0
Garun-Malam	G/Babba	49	5	10.2
Gaya	Gaya North	50	1	2.0
Gezawa	Mesa Tudu	50	1	2.0
Gwale	Ja’en	50	1	2.0
Gwarzo	Koya	48	2	4.2
Kabo	Durun	50	5	10.0
Kano Municipal	Sharada	50	4	8.0
Karaye	Turawa	39	4	10.3
Kibiya	Tarai	50	1	2.0
Kiru	Bauda	48	1	2.1
Kumbosto	Yan-Shana	50	2	4.0
Kunchi	Shuwaki	50	5	10.0
Kura	DaIiIi Kura	100	2	2.0
Madobi	Rikadawa	50	1	2.0
Makoda	Makoda city	50	3	6.0
Minjibir	Wasai	35	2	5.7
Nasarawa	K/Goji	50	11	22.0
Rano	Rano S/G	49	5	10.2
Rimin Gado	Rimin Gado	50	4	8.0
Rogo	Tajaye	42	4	9.5
Shanono	Marabutawa	50	4	8.0
Sumaila	SumaiIa	49	4	8.2
Takai	Faruruwa	50	12	24.0
Tarauni	Dantsinke	50	3	6.0
Tofa	Gajida	50	6	12.0
Tsanyawa	Tsanyawa	50	4	8.0
Tudun-Wada	Yar Yaso	50	6	12.0
Ungogo	Dankunkuru	50	6	12.0
Warawa	Jigawa	50	1	2.0
Wudil	Darki	50	2	4.0

### Circulating filarial antigen (CFA) survey

A serological survey was carried out following the WHO guidelines [12] in May 2015. Individuals living in the selected communities were sensitized and informed by community health workers and health officers of the urban LGAs about the survey. The sensitization was done in churches and mosques and other socio-economic groups. Additionally, a town crier was used in the selected communities to inform community members about the survey and individuals ≥5 years willing to take part in the survey were then invited to a designated community location (schools and urban health center) where finger-prick blood samples were taken. We used the immune-chromatography card test (ICT) (Alere, NOW, ICT filariasis kits; Binax, Portland, USA) for the detection of circulating filarial antigen in finger-prick blood samples taken during the day. All batches of ICT cards used were tested for quality control using serum from LF positive individuals, provided by the Noguchi Memorial Institute for Medical Research (NMIMR). All test results were read after 10 minutes according to the manufacturer’s instructions.

### Mosquito sampling

Mosquitoes were collected over a 4-month period from May to August 2015, using exit traps, gravid traps and pyrethrum knock-down spray sheet collections (PSC) in different households. The use of these various trapping methods was to augment the number of samples collected and analyzed for the study. The estimated sample size was 1000 vector mosquitoes (100 pools of 20 mosquitoes per pool per site) required in order to estimate an infection rate of 1% with a power of 0.80 [13]. Mosquito collections were done in the same communities where the parasitological surveys were undertaken. According to the information provided by the district health officers of the three urban communities, mosquito collection points were also selected based on the observation of risk factors such as presence of mosquito breeding sites, increasing potential exposure of the population to mosquito bites.

Firstly, exit (window) traps, targeting host-seeking adults were installed in fifteen different randomly selected households, five in each LGA. Householders were asked to collect mosquitoes for 10-days, each morning, during the mosquito collection period—for which they received a small remuneration. This yielded a total of 600 sampling days (3 LGAs x 5 traps x 10 days x 4 months). Both exit traps and PSC were performed in different households on the same day. However, when mosquito catches were consistently low in any household, such households were replaced by others in the neighborhood.

Pyrethrum spray catches (PSC) targeting resting adult mosquitoes were carried out inside the sleeping room of three randomly selected households (different from those used for the exit trap) in each site. Each month the collection period lasted for 7 days, therein yielding a total sampling effort of 252 sampling days (3 LGAs x 4 months x 7 days per month x 3 households per sampling day). A different set of three houses was sampled on each of the seven sampling days per month. The PSC method consisted of spraying an insecticide (pyrethroid) in selected rooms. Mosquitoes were collected on a white sheet covering the floor space of the sleeping room 10 minutes after spraying the rooms.

Gravid trap collections were performed outside five randomly selected collection points in each LGA. Each month the collection period lasted 7 days therein yielding a total sampling effort of 420 days (3 LGAs x 4 months x 7 days per month x 5 traps / site). Gravid traps are highly efficient for sampling *Culex* species. The trap attracts females with an oviposition attractant medium contained in a pan below the trap [14]. Previous studies in West African cities have shown that the mosquito fauna is dominated by *Culex* species [8,9,15], as a result of the polluted breeding environments. While *Culex* is considered as a non-vector species in West Africa [16], it has been shown that parasite DNA can be identified in both vector and non-vector species after ingestion of parasite positive blood [17,18]. As such, identifying *W*. *bancrofti* parasite or DNA in any mosquito species is enough to reveal the presence of an infected individual. The use of *Culex* gravid traps was therefore to augment the chances of identifying infected mosquitoes in the study areas.

### Mosquito processing

Each month, during the entomology survey mosquitoes collected by each trapping method were taken to the nearest suitable field laboratory for further processing. Mosquitoes collected were identified to the genus and species level using morphological identification keys [19,20]. Following identification, the specimens were placed individually in coded vials and kept dry in tins containing silica gel for future processing. The mosquitoes were then sent to the NTD Reference laboratory of the Noguchi Memorial Institute for Medical Research, for molecular identification and processing for *W*. *bancrofti* infection.

### Species and molecular forms of *Anopheles gambiae* complex

For the species identification of the members of the *An*. *gambiae* complex, a maximum of 100 mosquitoes were processed from each community. Where the number was less than 100, all mosquitoes were processed.

Genomic DNA was extracted from the legs of each mosquito, using the boiling preparation method; the legs were crushed in 100ml of distilled water and boiled at 95°C for 10 minutes. The supernatant was subsequently used as template for the PCR. Mosquitoes in the *An*. *gambiae* complex were then identified to species and molecular forms using the PCR method of Fanello et al. [21].

### Molecular identification of *W*. *bancrofti* DNA in mosquitoes

Mosquitoes were pooled according to community, trapping method and abdominal status. The pool range was 1–20. DNA was extracted from each pool using the Qiagen DNEasy tissue kit. The identification of *W*. *bancrofti* DNA was performed using the LAMP method [22] and the conventional PCR method [23], for confirmation. The LAMP method uses four primers that are simultaneously used to initiate DNA synthesis from the original unamplified DNA to generate a stem-loop DNA for subsequent LAMP cycling, and is thus faster, more sensitive and less susceptible to inhibition, compared to the conventional PCR method. We did not assess the infectivity rate of *W*. *bancrofti* stage specific L3 parasites. Only the presence of *W*. *bancrofti* DNA in mosquitoes was assessed, hence we cannot conclude on the status of transmission.

### Quality control

All assays included a known positive control (DNA extracted from a mosquito pool seeded with *W*. *bancrofti* microfilariae), and a negative control. All *W*. *bancrofti* positive pools were confirmed, by repeating the assay a second time. If a positive pool turn negative after repeat, the sample is re-run a third time for confirmation. If it is still negative, it is considered negative.

### Statistical analysis

The numbers of *Anopheles* and *Culex* mosquitoes were collated and calculated as percentages of the totals for site trapping method and month of collection. The pool screening software (Version 2.0) was used to estimate the maximum likelihood estimates (MLE) of the prevalence of *W*. *bancrofti* infection in the mosquitoes [24,25].

## Results

During this survey, 305, 304 and 372 individuals were examined for circulating filarial antigen (CFA) from Fagge, Ungogo and Nasarawa, respectively, giving a total of 981 individuals (Table 2). None of the subjects had detectable circulating *W*. *bancrofti* parasite antigen.

**Table 2:** ICT results for three urban LGAs of Kano state.

L.G.A(Community)	Sex	No. Tested	Age Group (years)					No. (%) +ve
			<11	11–20	21–30	31–40	>41	
Fagge (Jaba)	Females	167	41	45	34	23	24	0 (0.0)
	Males	138	32	51	27	11	17	0 (0.0)
Nasarawa (Gama)	Females	158	17	19	15	15	92	0 (0.0)
	Males	214	27	58	19	12	98	0 (0.0)
Ungogo (Dankunkuru)	Females	126	35	39	25	14	13	0 (0.0)
	Males	178	54	53	25	23	23	0 (0.0)
**Total**	**Females**	**451**	**93**	**103**	**74**	**52**	**129**	**0 (0.0)**
	**Males**	**530**	**113**	**162**	**71**	**46**	**138**	**0 (0.0)**

A total of 19,690 mosquitoes consisting of 2,600 *An*. *gambiae*, 17,082 *Culex* mosquitoes and 8 mosquitoes belonging to other species were collected (Table 3). The number of *Anopheles* mosquitoes collected was low in the dry season months of May, June and July, but peaked at the beginning of the rainy season in August.

**Table 3:** Monthly collections of mosquito species in the three urban L.G.A.s of Kano.

Month	Species	L.G.A. (Community)			Total
		Fagge (Jaba)	Nasarawa (Gama)	Ungogo (Dankunkuru)	
May	*An. gambiae*	2	0	21	25
	*Cx. quinquefascitus*	1257	2613	2312	6182
	*Other species*	0	0	1	1
June	*An. gambiae*	4	0	7	11
	*Cx. quinquefascitus*	435	628	144	1207
	*Other species*	0	0	2	2
July	*An. gambiae*	22	0	9	31
	*Cx. quinquefascitus*	684	820	101	1605
	*Other species*	0	0	0	0
August	*An. gambiae*	1304	76	1153	2533
	*Cx. quinquefascitus*	4545	1266	2277	8088
	*Other species*	2	1	2	5

A proportion of the mosquitoes were dissected to determine infection with *W*. *bancrofti*. In all, 8,809 mosquitoes were dissected- 1324 *An*. *gambiae* and 7,485 *Culex* mosquitoes. All mosquitoes dissected to examine for larvae of *W*. *bancrofti* were negative suggesting that transmission was not taking place. Molecular identification of *W*. *bancrofti* identification revealed the presence of DNA in both *An*. *gambiae* and *Culex* mosquitoes. 602 pools of mosquitoes (pool ranges of 1–20) were analyzed out of which 62 pools were positive for *W*. *bancrofti* DNA. Overall, higher prevalences were observed in *Anopheles* compared to *Culex* (Table 4). The prevalence of *W*. *bancrofti* DNA in Fagge was 0.69% in the *An*. *gambiae* and 0.64% in *Culex spp*. In Nasarawa, the prevalence of DNA in samples was 6.78% and 0.48% in *An*. *gambiae* and *Culex*, respectively, while in Ungogo it was 4.55% and 0.19% in *An*. *gambiae* and *Culex*, respectively. The data was also analyzed based on the trapping method used (Table 5). It can be observed that the use of the PSC and the gravid traps resulted in higher infection rates, compared to the used of the exit traps.

**Table 4:** *W*. *bancrofti* infection rates in pools of *Anopheles* and *Culex* mosquitoes by PCR examined from the study sites.

LGA	Species	Abdominal Condition	No. of mosquitoes	Pools examined	Pools positive	MLE (%)	95% CI
Fagge	*An*. *gambiae*	Unfed	311	17	1	0.33	0.01, 1.67
		Fed	192	14	2	1.04	0.12, 3.61
		Gravid	83	5	1	1.24	0.04, 6.24
		**Total**	**586**	**36**	**4**	**0.69**	**0.18, 1.77**
	*Culex spp*.	Unfed	730	42	2	0.28	0.03, 0.97
		Fed	265	19	3	1.21	0.23, 3.46
		Gravid	2636	141	17	0.69	0.38, 1.13
		**Total**	**3631**	**202**	**22**	**0.64**	**0.38, 0.99**
Nasarawa	*An*. *gambiae*	Unfed	20	2	0	-	-
		Fed	14	2	1	18.37	0.49, 93.84
		Gravid	3	2	1	42.27	1.60, 96.87
		**Total**	**37**	**6**	**2**	**6.78**	**0.81, 22.66**
	*Culex spp*.	Unfed	1396	73	8	0.61	0.24, 1.22
		Fed	365	24	2	0.57	0.07, 1.97
		Gravid	1463	81	5	0.35	0.11, 0.83
		**Total**	**3224**	**178**	**15**	**0.48**	**0.26, 0.82**
Ungogo	*An*. *gambiae*	Unfed	302	18	9	3.96	1.69, 7.68
		Fed	84	8	3	4.86	0.94, 14.11
		Gravid	38	4	2	12.55	1.32, 55.99
		**Total**	**424**	**30**	**14**	**4.55**	**2.34, 7.85**
	*Culex*. *spp*	Unfed	1472	78	3	0.21	0.04, 0.60
		Fed	497	32	0	-	-
		Gravid	657	40	2	0.31	0.04, 1.08
		**Total**	**2626**	**150**	**5**	**0.19**	**0.06, 0.46**

**Table 5:** *W*. *bancrofti* infection rates in pools of *Anopheles* and *Culex* mosquitoes by trapping method.

LGA	Species	Method	No. of mosquitoes	Pools examined	Pools positive	MLE (%)	95% CI
Fagge	*An*. *gambiae*	Exit Trap	346	20	1	0.29	0.009, 1.47
		*Cx*. Gravid Trap	13	1	1	-	-
		Pyrethrum Spray	227	15	2	0.89	0.11, 3.09
		**Total**	**586**	**36**	**4**	**0.69**	**0.18, 1.77**
	*Culex spp*.	Exit Trap	311	21	1	0.33	0.01, 1.67
		*Cx*. Gravid Trap	3056	161	20	0.69	0.40, 1.10
		Pyrethrum Spray	264	20	1	0.38	0.01, 1.95
		**Total**	**3631**	**202**	**22**	**0.64**	**0.38, 0.99**
Nasarawa	*An*. *gambiae*	Exit Trap	19	1	0	-	-
		*Cx*. Gravid Trap	0	-	-	-	-
		Pyrethrum Spray	18	5	2	24.49	2.69, 74.99
		**Total**	**37**	**6**	**2**	**6.78**	**0.81, 22.66**
	*Culex spp*.	Exit Trap	25	7	0	-	-
		*Cx*. Gravid Trap	2841	147	12	0.44	0.21, 0.79
		Pyrethrum Spray	358	24	3	0.87	0.17, 2.51
		**Total**	**3224**	**178**	**15**	**0.48**	**0.26, 0.82**
Ungogo	*An*. *gambiae*	Exit Trap	326	20	11	4.63	2.16, 8.50
		*Cx*. Gravid Trap	14	4	0	-	-
		Pyrethrum Spray	84	6	3	5.44	1.04, 16.10
		**Total**	**424**	**30**	**14**	**4.55**	**2.34, 7.85**
	*Culex*. *spp*	Exit Trap	737	26	0	-	-
		*Cx*. Gravid Trap	1605	90	4	0.25	0.06, 0.65
		Pyrethrum Spray	284	34	1	0.17	0.005, 0.89
		**Total**	**2626**	**150**	**5**	**0.19**	**0.06, 0.46**

Molecular analysis of samples of the *An*. *gambiae s*.*l*. showed that most members of the species complex in the three study communities were *An*. *coluzzii*, previously known as the M form of *An*. *gambiae s*.*s*. Only one *An*. *gambiae* s.s. and one *An*. *arabiensis* were identified (Table 6).

**Table 6:** Distribution of members of the *An*. *gambiae* complex in the study sites.

LGA	No. analyzed	*An*. *coluzzii* (M Form)	*An*. *gambiae* s.s (S Form)	M/S	*An*. *arabiensis*
Fagge	100	99	0	0	1
Nasarawa	35	34	1	0	0
Ungogo	100	99	0	1	0

## Discussion

A cross-sectional ICT antigen detection survey carried out in May 2015 in the three urban LGAs of Kano state revealed that none of the 981 individuals aged ≥5 years had detectable levels of CFA. This contradicts the mapping results undertaken by the NTD program from the Federal Ministry of Health in the study area in 2010 (5 years earlier) which reported prevalence of circulating filarial antigen varying between 2% and 12% in these LGAs. It will be important to know what factors have led to the reduction in LF prevalence from the mapping records or if the ICT tests available were recording false positives. It is known that vector control interventions have the potential to impact LF elimination activities [26,27] and the level of vector control interventions implemented between previous mapping and the current study could provide further evidence for LF elimination using vector control in Africa. In Kano state free distribution of insecticide treated nets was reported, which showed ITN ownership increasing more than 10-fold, from 6% to 71% [28]. It is further possible that the insecurity in northern Nigeria could have contributed significantly to the increase in the number of people migrating from rural areas to cities such as Kano. 3.3 million persons were displaced due of Boko Haram attacks from Borno, Yobe and Adamawa States to Kano in the last 5 years [29]. As a result it may be possible that the LF prevalence in urban Kano in 2010 may be a result of rural to urban migration of antigen positive individuals that are likely to have acquired the infection elsewhere.

The two main mosquito species observed in the study sites were *Culex quinquefasciatus* and *An*. *gambiae s*.*l*. The higher proportion of *An*. *coluzzii* (formally the M form of *An*. *gambiae*) could be explained by the fact that these prefer ephemeral breeding sites, found more in urban and arid areas compared to *An*. *gambiae s*.*s* [30].

The study demonstrated the presence of *An*. *gambiae s*.*l* (principally *An*. *coluzzi*) and *Culex quinquefasciatus* specimens containing DNA of *W*. *bancrofti* in urban settings of Kano state. The prevalence of *W*. *bancrofti* DNA in *Culex* species from Fagge, Nasarawa and Ungogo LGAs were low (≤0.65%, 0.48% and 0.19% respectively) compared to *An*. *gambiae* in which 0.96%, 6.78% and 4.55% DNA positivity were recorded respectively. Despite these levels of *W*. *bancrofti* DNA prevalence, we are unable to confirm transmission by *An*. *gambiae* and *Culex quinquefasciatus* as vectors of *W*. *bancrofti* in Kano because the detection of parasite DNA in mosquitoes does not necessarily imply that LF transmission is ongoing in a given setting. Studies have shown that mosquitoes that fed on people with very low levels of MF sometimes ingest MF but rarely produced infective larvae [27]. In Fagge LGA, a low DNA detection level (0.69%) was recorded after processing 586 specimens of *An*. *gambiae s*.*l*. To confirm on-going LF transmission in Ungogo LGA where a high infection rate of *W*. *bancrofti* has been reported within *An*. *gambiae* species, the use of newer generation diagnostic tools, such as Wb123 [31], might prove useful in the identification of current and active transmission in the untreated population.

Similar to other investigations elsewhere [9,32], this study does point to the fact that molecular xenomonitoring methods are superior to parasitological surveys in humans. Nonetheless, defining the elimination thresholds using molecular xenomonitoring requires defining at which prevalence estimate, in mosquitoes, transmission can be said to be interrupted. The observed prevalence of parasite DNA in the mosquitoes fall within the cut-off points of 0.25%, 0.5% and 1% suggested for *Culex* areas [33–35], and 0.65% and 1.0% for *Anopheles* areas [13,36]. However, these cut-offs are only suggestive. The challenge therefore is to clearly define the elimination thresholds in vectors considering, vector control activities, vector species and abundance, biting rates etc.

A limitation to this survey was the inability to attain the required sample sizes, and as such, the wide confidence intervals, especially in the *Anopheles* collections indicate the need for much larger sample sizes. This could be due to the period of mosquito collection, as the mosquitoes were collected mostly during the dry season. As such the timing of xenomonitoring surveys, barring programmatic difficulties, is very important to make meaningful assessments. While *Anopheles* species are considered the major vectors of *W*. *bancrofti* in West Africa, earlier studies in rural settings of Kano State revealed L3 *W*. *bancrofti* infection in *Culex quinquefasciatus* [11]. Other studies elsewhere in Nigeria also revealed high infection and infectivity rates in *Culex* mosquitoes [37,38]. The identification of DNA of the parasite in both mosquito genera goes to lend support to these earlier findings. Further, *An*. *coluzzii* is believed to be a more efficient vector of LF compared to *An*. *gambiae* s.s [39,40], and the predominant nature of the former could imply a more efficient transmission of LF in the study locations. However, the promotion of environmental sanitation, coupled with vector control measures would go a long way in reducing LF infection and transmission. It is note-worthy that while the parasitological survey did not detect any infection, entomological surveys targeted at mosquito breeding sites identified infections, indicating these as being more sensitive in detecting any ongoing transmission. The parasitological surveys would also have identified infections in the community, if these were targeted at high-risk individuals such as those living in the vicinity of those breeding sites. Future assessments should therefore aim at identifying such individuals and stratifying the study communities so as to improve the outcome of parasitological surveys.

The higher local prevalence of infection observed in the mosquitoes, compared to lower aggregated values may reflect the spatial heterogeneity and clustering of LF prevalence and transmission [41,42]. Analyzing disaggregated data to the trap level (especially if the coordinates of trapping sites were taken) might have been useful in identifying focal areas of transmission, considering that mosquitoes have limited flight ranges. This study also reveals the utility of analyzing mosquitoes that have recently fed in determining mosquito infection prevalence [13]. As can be seen from Table 4, fed and gravid mosquitoes generally had higher infection levels compared to unfed mosquitoes. While some pools of unfed mosquitoes were found positive, parity dissection were not done in this study. This is because the mosquitoes sent to the NMIMR were stored dry on silica gel, and parity dissections are best done on fresh samples. Thus, the positive unfed mosquitoes may represent parous mosquitoes. Filaria DNA can be detected in mosquitoes that have processed *W*. *bancrofti* infected blood [43] and it is therefore important that molecular xenomonitoring studies aimed at analyzing unfed mosquitoes, limit these analyses to parous mosquitoes in order to minimize cost. Further the analysis based on the trapping methods (Table 5) reveal that trapping of indoor resting mosquitoes using the PSC and gravid mosquitoes may be better tools in xenomonitoring than exit traps.

Assessing *W*. *bancrofti* DNA within mosquito specimens is an indicator of the presence of infected humans and possible ongoing transmission [41,44]. However, confirming the transmission of LF requires the identification of infective stage larvae either through molecular methods or through dissection. While our results revealed widespread presence of *W*. *bancrofti* DNA in the mosquito population, we were unable to find a single positive mosquito given the number of mosquitoes dissected. Even though PCR is far more sensitive than dissection [13], a limitation of this study is that the mosquitoes dissected for *W*. *bancrofti* larvae were not also subjected to the more sensitive molecular analysis. Such mosquitoes were teased, examined and discarded, and only un-dissected mosquitoes were shipped to the NMIMR for analyses. Additionally, assessing *W*. *bancrofti* DNA within mosquitoes is not a good indicator to confirm the presence of L3 larvae of LF parasite. But due to financial and logistic challenges related to assessment of the infectivity–L3 –rate (because mosquito specimens should be stored with RNA processed through real time PCR technique) we were not able to evaluate the infectivity rate. Molecular xenomonitoring, however, proved more effective in this study and has also been shown to be a promising tool in post-MDA surveillance [32,33].

In conclusion, despite the fact that infection of *W*. *bancrofti* DNA within *Anopheles gambiae* specimens was above 1% in some areas, the decline in antigen prevalence from the 2010 survey to the current survey led to the conclusion that LF transmission is not sustainable in the three urban LGAs of Kano state. While the mosquito biting rate could not be determined in this study, it is believed that the low numbers of *An*. *gambiae* collected is not sufficient to sustain transmission, as studies suggest that 2700 to over 100,000 infective bites are required for a new infection to be established [45]. The increased use and coverage of insecticide treated nets [28], and other activities such as mosquito-proofing houses with screens and ceilings [46], will lead to a reduction in the indoor densities of mosquitoes. These in turn will further decrease the vector-human contact in these urban areas, and ultimately the inefficient transmission of the parasite by mosquitoes. Based on these we could not recommend MDA. However, the Federal Ministry of Health advised that two rounds of MDA should be undertaken. The first round of MDA was undertaken in 2016. This study demonstrated that entomological surveys are probably more sensitive indicators of the presence of infection. However, we could not demonstrate evidence for active transmission as we did not assess the infectivity rate for technical reasons. While no individual was found positive for antigen in the sample population surveyed, it is possible that antigen positive and potentially microfilaremic adults could potentially be contributing to transmission, especially since positive mosquitoes were detected. The use of the wb123 antibody assays may provide further evidence to the presence or absence of active transmission. Re-assessing the prevalence of *W*. *bancrofti* infection (both in the human and mosquito population) after a couple of years is recommended.
